# Association between triage level and outcomes at Médecins Sans Frontières trauma hospital in Kunduz, Afghanistan, 2015

**DOI:** 10.1136/emermed-2020-209470

**Published:** 2021-11-10

**Authors:** Hadjer Latif Daebes, Linnea Latifa Tounsi, Maximilian Nerlander, Martin Gerdin Wärnberg, Momer Jaweed, Bashir Ahmad Mamozai, Masood Nasim, Miguel Trelles, Johan von Schreeb

**Affiliations:** 1 Center for Research on Health Care in Disasters, Department of Global Public Health, Karolinska Institutet, Stockholm, Sweden; 2 Function Perioperative Medicine and Intensive Care, Karolinska University Hospital, Solna, Sweden; 3 Medical Department, Médecins Sans Frontières, Kunduz, Afghanistan; 4 Medical Coordination, Médecins Sans Frontières, Kabul, Afghanistan; 5 Medical Department, Operational Centre Brussels, Doctors without Borders, Bruxelles, Belgium

**Keywords:** emergency care systems, emergency department, global health, Trauma, triage

## Abstract

**Background:**

Five million people die annually due to injuries; an increasing part is due to armed conflict in low-income and middle-income countries, demanding resolute emergency trauma care. In Afghanistan, a low-income country that has experienced conflict for over 35 years, conflict related trauma is a significant public health problem. To address this, the non-governmental organisation Médecins Sans Frontières (MSF) set up a trauma centre in Kunduz (Kunduz Trauma Centre (KTC)). MSF’s standardised emergency operating procedures include the South African Triage Scale (SATS). To date, there are few studies that assess how triage levels correspond with outcome in low-resource conflict settings

**Aim:**

This study aims to assess to what extent SATS triage levels correlated to outcomes in terms of hospital admission, intensive care unit (ICU) admission and mortality for patients treated at KTC.

**Method and materials:**

This retrospective study used routinely collected data from KTC registries. A total of 17 970 patients were included. The outcomes were hospital admission, ICU admission and mortality. The explanatory variable was triage level. Covariates including age, gender and delay to arrival were used. Logistic regression was used to study the correlation between triage level and outcomes.

**Results:**

Out of all patients seeking care, 28.7% were triaged as red or orange. The overall mortality was 0.6%. In total, 90% of those that died and 79% of ICU-admitted patients were triaged as red.

**Conclusion:**

The risk of positive and negative outcomes correlated with triage level. None of the patients triaged as green died or were admitted to the ICU whereas 90% of patients who died were triaged as red.

**Key messages**

**What is already known on this subject**

- During 2015 approximately 18 300 patients sought help at the Médecins Sans Frontières trauma centre in Kunduz (Kunduz Trauma Centre (KTC)). To help prioritise treatment KTC used the South African Triage Scale. To date, there are few studies that assess how triage levels correspond with outcome in low-resource conflict settings.

**What this study adds**

- Our study suggests that triage levels had high sensitivity to predict outcome. Delay in arrival and low mortality indicate that prehospital management is key in this setting to increase trauma survivability.

## Background

An estimated five million people die from injuries every year, disproportionately affecting low-income and middle-income countries (LMICs).^1^ It is estimated that a reduction in the trauma mortality rate of LMICs to that of high-income countries would prevent approximately two million deaths annually.^2 3^ Violence and conflict make up an increasing proportion of the global burden of injury.^4^ Globally, more than 1 in 10 violent deaths occur in conflict settings.^5^

In Afghanistan, a low-income country affected by war and political violence for more than 35 years, conflict-related trauma is a significant health problem.^6 7^ The country is among the top five countries in the world affected by violent deaths due to conflict, killing nearly 15 000 people annually and accounting for 5.9% of all annual deaths.^8^ The northern province of Kunduz is a region particularly affected by conflict.^5 8–10^ In order to meet trauma care needs Médecins Sans Frontières (MSF) opened Kunduz Trauma Centre (KTC) in 2011. This facility was operational until 3 October 2015, when a US airstrike hit and destroyed the facility, killing 14 MSF staff, 24 patients and 4 caretakers.^10^

Care at dedicated trauma centres reduces trauma-related mortality by 9%–25% compared with non-trauma centres.^11^ A central mechanism of trauma care is to triage patients in order to prioritise resources and ensure fast identification of potentially fatal conditions of those arriving to the hospital ED.^12^ MSF uses standardised ED packages to triage and provide appropriate initial care to trauma patients at its trauma facilities.^13^ The South African Triage Scale (SATS) is the main triage system that is used in the MSF ED packages. While numerous studies evaluate triage systems, few assess how triage levels correspond to outcomes in low-resource armed conflict settings. This study therefore aims to assess to what extent SATS triage levels correlated to outcomes in terms of hospital admission, intensive care unit (ICU) admission and mortality for patients treated at KTC.

## Materials and methods

KTC was located in Kunduz city, the fifth largest city in Afghanistan situated in Kunduz province, with an estimated population of one million.^14^ Before the opening of KTC, patients had to travel to Kabul, approximately 340 km by road, or to Pakistan, for trauma care. During its functional years, the trauma centre offered free around-the-clock care, performed more than 15 000 surgeries and treated more than 68 000 emergency patients with a staff of 460 employees.^10^

**Figure 1:**
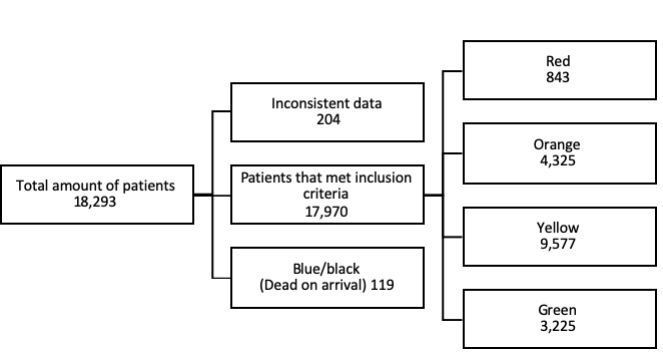
Study sample. Patients admitted to Kunduz Trauma Centre (KTC) January–September 2015.

This study included all patients registered at the ED at KTC from 1 January 2015 to 18 September 2015. The study period was chosen as data from other years was inconclusive and did not provide variables of sufficient quality for this study. This study included all 18 293 patients that were registered at the ED during the study period. Out of these, 323 patients were excluded due to inconsistent data in key variables and due to being registered as dead on arrival (figure 1).

The data used in this study were routinely collected using a standardised data collection form used across all MSF missions. Data were recorded in a logbook and then transferred into an electronic database (Microsoft Excel). The database was transmitted to the MSF headquarters in Brussels where it was reviewed for accuracy. Discrepancies were addressed and corrected after contact with programme personnel involved in data entry.

SATS was the triage system used during normal activities at KTC. It is based on physiological parameters and a list of clinical discriminators. Each of the five triage levels provide time-to-treatment targets; red (immediately), orange (less than 10 min), yellow (less than 1 hour), green (less than 4 hours) and blue (dead on arrival) (online supplemental figure 1). The MSF ED package also includes the Simple Triage and Rapid Treatment triage system which was used in case of multiple casualty incidents. It assesses the ability to walk, spontaneous breathing, respiratory rate, perfusion and level of consciousness.^15^ The majority of patients at KTC were triaged using SATS as this was the system used during normal activities at the ED. Hence our study only assessed SATS. The data set used includes variables to ascertain which system was used for a given patient but is not consistently recorded.

10.1136/emermed-2020-209470.supp1 Supplementary data

Hospital admission, ICU admission and mortality were chosen as outcomes, serving as proxy measures for injury severity. Patients admitted to the ICU were a subgroup of those admitted to hospital. The explanatory variable was triage level. Covariates including age, sex and delay to arrival were used to characterise the study sample and to adjust the association between triage levels and outcome.

Statistical analysis was performed using R V.3.3.2. (The R Foundation, R Core Team, 1020 Vienna, Austria). Descriptive statistics are reported as count and percentage, mean and SD or median and IQR as applicable. First an unadjusted analysis using logistic regression was conducted for each outcome. In terms of ICU admission and mortality the analysis only included patients triaged as red or orange. Second, an adjusted analysis using logistic regression that included the covariates sex, age and delay to arrival in addition to triage level was conducted. We conducted the adjusted analysis to account for factors not included in the triage systems. We used 95% CIs and a significance level of 5%.

### Patient and public involvement

Patients were not informed about this specific study since it is a retrospective study and KTC could provide no follow-up. Therefore, patient and public involvement were not applicable.

## Results

Data were captured on a total of 18 293 patients out of which 323 were excluded. Thus, a total of 17 970 patients were included in the final analysis. Out of all patients 843 (4.7%) were triaged as red, 4325 (24%) as orange, 9577 (53.3%) as yellow and 3225 (17.9%) as green (figure 1).

Out of all patients included 76.4% were men (table 1). The mean patient age was 23.1 (SD 16.8) years and 61.9% of patients were under 25 years of age. Patients categorised as red had a higher mean age (25.1, SD 15.8) while age was lower in all other triage categories (p<0.001).

**Table 1:** Patient characteristics for patients admitted at the Kunduz Trauma Centre January–September 2015

	Red	Orange	Yellow	Green	Total
Total	843	4325	9577	3225	17 970
Male (%)	719 (85.3)	3524 (81.5)	7134 (74.5)	2351 (72.9)	13 728 (76.4)
Age category, years (%)					
<5	42 (5.0)	392 (9.1)	1133 (11.8)	209 (6.5)	1776 (9.9)
5–9	83 (9.8)	506 (11.7)	1270 (13.3)	163 (5.1)	2022 (11.3)
10–14	103 (12.2)	551 (12.7)	1552 (16.2)	352 (10.9)	2558 (14.2)
15–19	102 (12.1)	488 (11.3)	1304 (13.6)	632 (19.6)	2526 (14.1)
20–24	117 (13.9)	576 (13.3)	1009 (10.5)	520 (16.1)	2222 (12.4)
25–29	102 (12.1)	459 (10.6)	725 (7.6)	349 (10.8)	1635 (9.1)
30–34	77 (9.1)	291 (6.7)	506 (5.3)	233 (7.2)	1107 (6.2)
35–39	71 (8.4)	254 (5.9)	428 (4.5)	185 (5.7)	938 (5.2)
40–44	37 (4.4)	217 (5.0)	378 (3.9)	162 (5.0)	794 (4.4)
45–49	30 (3.6)	143 (3.3)	300 (3.1)	116 (3.6)	589 (3.3)
50–54	23 (2.7)	143 (3.3)	337 (3.5)	122 (3.8)	625 (3.5)
55–59	17 (2.0)	63 (1.5)	145 (1.5)	52 (1.6)	277 (1.5)
60–64	21 (2.5)	115 (2.7)	219 (2.3)	72 (2.2)	427 (2.4)
65–69	4 (0.5)	40 (0.9)	86 (0.9)	15 (0.5)	145 (0.8)
70–74	8 (0.9)	56 (1.3)	100 (1.0)	32 (1.0)	196 (1.1)
75–79	2 (0.2)	11 (0.3)	31 (0.3)	3 (0.1)	47 (0.3)
80–84	0 (0.0)	9 (0.2)	35 (0.4)	5 (0.2)	49 (0.3)
85+	4 (0.5)	11 (0.3)	19 (0.2)	3 (0.1)	37 (0.2)
Mean age (SD)	25.13 (15.77)	23.92 (16.98)	21.94 (17.24)	24.90 (15.15)	23.10 (16.80)
Multiple injuries (%)	269 (31.9)	567 (13.1)	247 (2.6)	39 (1.2)	1122 (6.2)
Delay to arrival (%)					
≤1 hour	348 (41.3)	1431 (33.1)	2149 (22.4)	398 (12.3)	4326 (24.1)
≤6 hours	299 (35.5)	1095 (25.3)	1131 (11.8)	204 (6.3)	2729 (15.2)
≤1 day	161 (19.1)	1332 (30.8)	4258 (44.5)	1430 (44.3)	7181 (40.0)
>1 day	35 (4.2)	467 (10.8)	2039 (21.3)	1193 (37.0)	3734 (20.8)
Nature of injury (%)					
Burn	2 (0.2)	46 (1.1)	81 (0.8)	13 (0.4)	142 (0.8)
Contusion or superficial	26 (3.1)	771 (17.8)	3646 (38.1)	2037 (63.2)	6480 (36.1)
Dislocation	0 (0.0)	41 (0.9)	49 (0.5)	4 (0.1)	94 (0.5)
Fracture	338 (40.1)	1930 (44.6)	3841 (40.1)	439 (13.6)	6548 (36.4)
Internal organ injury	191 (22.7)	129 (3.0)	47 (0.5)	20 (0.6)	387 (2.2)
Open wound	158 (18.7)	648 (15.0)	676 (7.1)	125 (3.9)	1607 (8.9)
Others non-trauma	6 (0.7)	47 (1.1)	137 (1.4)	73 (2.3)	263 (1.5)
Others trauma related	119 (14.1)	691 (16.0)	1002 (10.5)	477 (14.8)	2289 (12.7)
Sprains and strains	0 (0.0)	14 (0.3)	59 (0.6)	29 (0.9)	102 (0.6)
Unclassified	3 (0.4)	8 (0.2)	39 (0.4)	8 (0.2)	58 (0.3)
Self-referred (%)	835 (99.1)	4323 (100.0)	9577 (100.0)	3225 (100.0)	17 960 (99.9)
Admission (%)	708 (84.0)	1856 (42.9)	1029 (10.7)	84 (2.6)	3677 (20.5)
Intensive care unit mission (%)	216 (25.6)	51 (1.2)	6 (0.1)	0 (0.0)	273 (1.5)
Died (%)	90 (10.7)	10 (0.2)	0 (0.0)	0 (0.0)	100 (0.6)

A total of 3677 (20.5%) patients admitted to the ED were hospitalised, the majority of whom were triaged as either orange (1,856) or yellow (1,029). Out of all the patients admitted to hospital 273 (1.5%) patients were admitted to ICU, 97.8% of whom were triaged as red or orange. Altogether 25.6% of all red patients were admitted to the ICU. A total of 100 (0.6%) patients died, 90% of whom were triaged as red, the remaining 10 as orange. The most common site for injury for all triage levels was the extremities, affecting 84% of patients.

The risk of hospital admission increased with each triage level (table 2).

**Table 2:** Associations of triage levels with hospital admission

	OR	95% CI lower bound	95% CI upper bound	P value
Red (reference)	1			
Orange	0.143	0.118	0.173	<0.05
Yellow	0.023	0.019	0.028	<0.05
Green	0.005	0.004	0.007	<0.05

Both early (≤6 hours) and delayed arrival (>1 day) showed an increased risk for hospital admission (table 3).

**Table 3:** Adjusted associations of triage levels with hospital admission

	OR	95% CI lower bound	95% CI upper bound	P value
Red (reference)	1			
Orange	0.141	0.116	0.171	<0.05
Yellow	0.023	0.019	0.028	<0.05
Green	0.005	0.003	0.006	<0.05
Male gender	1.187	1.069	1.320	<0.05
Age	1.006	1.003	1.008	<0.05
Delay to arrival ≤1 hour (reference)	1			
≤6 hours	2.167	1.914	2.454	<0.05
>6 hours to ≤24 hours	1.032	0.920	1.157	0.593
>1 day	2.021	1.768	2.310	<0.05

Both the risk of ICU admission and death increased for red triage levels in comparison to orange triage levels (tables 4 and 5). For ICU admission red triage level had a significantly higher risk.

**Table 4:** Associations of triage levels with intensive care unit admission

	OR	95% CI lower bound	95% CI upper bound	P value
Red (reference)	1			
Orange	0.035	0.025	0.047	<0.05

**Table 5:** Associations of triage levels with mortality

	OR	95% CI lower bound	95% CI upper bound	P value
Red	1			
Orange	0.019	0.009	0.036	<0.05

Early arrival (≤6 hours) showed an increased risk for ICU admission in the orange and red triage levels (table 6).

**Table 6:** Adjusted associations of triage levels with intensive care unit admission

	OR	95% CI lower bound	95% CI upper bound	P value
Red (reference)	1			
Orange	0.032	0.023	0.044	<0.05
Male gender	1.100	0.757	1.630	0.624
Age	0.975	0.965	0.985	<0.05
Delay to arrival ≤1 hour (reference)	1			
≤6 hours	1.882	1.361	2.613	<0.05
>6 hours to ≤24 hours	1.462	0.999	2.134	0.049
>1 day	1.544	0.809	2.793	0.167

Neither gender, age nor delay to arrival showed any significant effect on mortality for red and orange triage level patients (table 7).

**Table 7:** Adjusted associations of triage levels with mortality

	OR	95% CI lower bound	95% CI upper bound	P value
Red (reference)	1			
Orange	0.019	0.009	0.036	<0.05
Male gender	1.243	0.685	2.453	0.500
Age	1.019	1.006	1.031	<0.05
Delay to arrival ≤1 hour (reference)	1			
≤6 hours	1.044	0.639	1.697	0.863
>6 hours to ≤24 hours	1.134	0.641	1.961	0.659
>1 day	1.011	0.333	2.512	0.982

## Discussion

This study confirmed a negative correlation between SATS triage levels and hospital admission, ICU admission and in-hospital death. Hospital admission increased with every triage level and red patients had a considerably higher risk of ICU admission in comparison to orange patients. Likewise, red patients had a significantly higher risk of mortality. Our results suggest triage levels applied at the KTC had high sensitivity to predict severity outcome for high-risk trauma patients in this context. This is one of few studies to study the associations between triage level and outcomes in a low-resource conflict setting.

Similar results have been shown by Dalwai *et al* 16 showing the validity of SATS at KTC having mortality and hospital admission as outcomes. It concluded that SATS can determine the accuracy of triage levels applied to the most vulnerable and ill patients. Our study considers ICU admission as a severity outcome, thus further indicating the validity of the use of SATS at KTC. The validity and reliability of a triage scale can be calculated using different means. SATS has been validated by the comparison of different triage-level outcomes depending on who performs the triage.^17^ It is also possible to validate the triage scale by comparing it to another validated triage scale or by evaluating each triage level to predicting outcome. Farrokhnia *et al* have researched the validity and reliability of multiple ED triage scales.^18^ They found that most triage scales had insufficient reliability in comparison to each other or to a gold standard. The study also revealed a lack of validity in predicting hospital mortality and admission. None of the studies included in the systematic review adjusted mortality rate to age and gender. In our study we have chosen to study the association between triage level and outcome. Although the data limited the possibility to evaluate the triage level to a gold standard, our methods could still estimate the reliability and validity of the triage levels.

Although it is not surprising that SATS as a triage scale could predict negative outcome, it is interesting to find that similar results were shown even after results were adjusted to covariates. In our study only 84 (2.6%) patients triaged as green were hospitalised, none of whom were admitted to the ICU or died, which indicates high specificity. However, both early (≤6 hours) and delayed arrival (>1 day) showed an increased risk for hospital admission in all triage levels while early arrival (≤6 hours) showed an increased risk for ICU admission in orange and red triage levels. These results indicate that delay correlates to the risk of negative outcome.

Only 100 patients died in the ED, corresponding to a mortality rate of 0.6%, while 2.7% of hospitalised patients died. This is a considerably lower mortality compared with trauma centres in USA that report rates around 8%.^11^ Four public hospitals in Mumbai, Kolkata and New Delhi in India, had a mortality rate of 7.3% within the first 24 hours.^19^ This suggests that the mortality rate of KTC was remarkably low, which may imply that the most critically ill patients did not reach the hospital in time for care due to gaps in prehospital care capacity and capability. This was suggested as a reason for similarly low hospital mortality rates in a study from Iraq during the Mosul offensive.^20 21^

A key factor to reduce mortality is early hospital care. Our study shows that almost all patients (99.9%) were self-referred to the hospital, which not only causes delay in treatment but indicates yet again that the most critically ill patients most likely never reached the trauma facility. This inevitably increases the prehospital mortality rate. An American study conducted on US troops deployed to Iraq and Afghanistan showed that approximately 85% of prehospital deaths were due to haemorrhage, which indicates that early arrival is key for survival and that a high proportion of trauma deaths may be preventable with rapid transport to hospital.^22^ A study conducted by Guzmán *et al* shows that 25.5% of trauma patients arrived with a >24 hours delay at KTC during 2013–2015, indicating the need of increased access to prehospital care and transport in the area.^23^ In our study, only 7055 (39.3%) of patients reached care within 6 hours, and a fourth of patients within 1 hour, although, the patients triaged as red had the least delay. Delay is an obstacle to the paradigm of early and fast treatment of patients and should be minimised to further improve survivability.^22 24^

Targeted public health action to strengthen implementation of triage systems and well-organised emergency care improve trauma survivability and are all a part of a resolution from the WHO’s seventy-second World Health Assembly on Emergency and Trauma care (WHA72/31). Our study, as well as a study by Hemat *et al* concludes that KTC saved many lives and that the majority of patients at KTC were successfully treated.^25^ It is estimated that more than 154 250 disability-adjusted life years were averted at KTC.^26^ The study also concludes that there was a strong need of implementation of prehospital care. Follow-up studies on long-term disability for patients in correlation to their initial triaging could give a wider perspective regarding the importance of correctly applied triage levels and possibly also a wider knowledge on which aspects to take into consideration while triaging.

### Limitations

The broad inclusion criteria might have had a negative impact on the significance of the results, yet it was considered necessary to maintain a heterogeneous population for the results to be representative. The US bombing of the KTC and the subsequent destruction of medical records limited the patient follow-up.

## Conclusion

This study confirmed a negative correlation between SATS triage level and hospital admission, ICU-admission and in-hospital death. Our results suggest triage levels applied at the KTC had high sensitivity to predict severity outcome for high-risk trauma patients. Our results indicate that delay in arrival had an impact on triage levels. Further, the low mortality found at KTC indicates that many critically injured trauma patients in Kunduz may not have survived long enough to reach care. Therefore, in this setting, targeted efforts to improve prehospital management and transport is likely to improve both triage level outcomes and have a positive impact on trauma survivability.

## Data Availability

Data are available upon reasonable request. Data may be obtained from a third party and are not publicly available.
