# Supplementary figures and images for: Association between triage level and outcomes at Médecins Sans Frontières trauma hospital in Kunduz, Afghanistan, 2015
Source: Emerg Med J. 2021 Nov 10;39(8):628–33. doi: 10.1136/emermed-2020-209470 (PMC9304096; doi:10.1136/emermed-2020-209470)

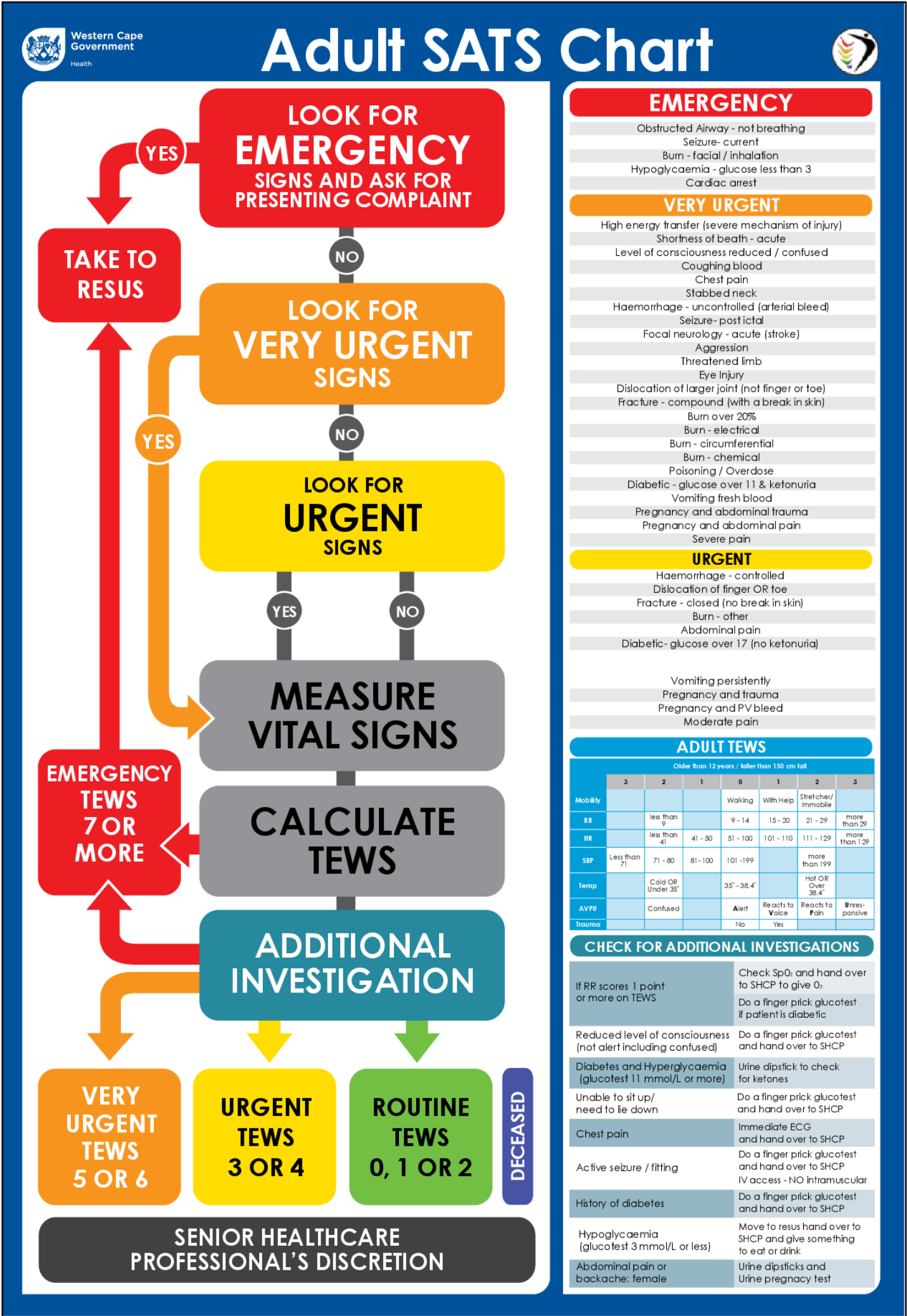

Supplement: Supplementary data [file emermed-2020-209470supp001.pdf]
